# Prevalence of Arm Weakness, Pre-Stroke Outcomes and Other Post-Stroke Impairments Using Routinely Collected Clinical Data on an Acute Stroke Unit

**DOI:** 10.1177/15459683241229676

**Published:** 2024-02-10

**Authors:** Emily J. Dalton, Rebecca Jamwal, Lia Augoustakis, Emma Hill, Hannah Johns, Vincent Thijs, Kathryn S. Hayward

**Affiliations:** 1 Occupational Therapy Department, Austin Health, Heidelberg, VIC, Australia; 2 Department of Physiotherapy, University of Melbourne, Parkville, VIC, Australia; 3 Melbourne Medical School, University of Melbourne, Melbourne, VIC, Australia; 4 Florey Institute of Neuroscience and Mental Health, University of Melbourne, Heidelberg, VIC, Australia; 5 Department of Neurology (Austin), Austin Health, Heidelberg, VIC, Australia; 6 Department of Medicine, University of Melbourne, Parkville, VIC, Australia; 7 Departments of Physiotherapy, Medicine (RMH), and Florey Institute of Neuroscience and Mental Health, University of Melbourne, Carlton, VIC, Australia

**Keywords:** stroke rehabilitation, upper extremity, clinical frailty, function

## Abstract

**Introduction:**

The prevalence of upper limb motor weakness early post-stroke may be changing, which can have clinical and research implications. Our primary aim was to describe the prevalence of upper limb motor weakness early post-stroke, with a secondary aim to contextualize this prevalence by describing pre-stroke outcomes, other post-stroke impairments, functional activities, and discharge destination.

**Methods:**

This cross-sectional observational study extracted clinical data from confirmed stroke patients admitted to a metropolitan stroke unit over 15-months. The primary upper limb weakness measure was Shoulder Abduction and Finger Extension (SAFE) score. Demographics (eg, age), clinical characteristics (eg, stroke severity), pre-stroke outcomes (eg, clinical frailty), other post-stroke impairments (eg, command following), functional activities (eg, ambulation), and discharge destination were also extracted.

**Results:**

A total of 463 participants had a confirmed stroke and SAFE score. One-third of patients received ≥1 acute medical intervention(s). Nearly one-quarter of patients were classified as frail pre-stroke. Upper limb weakness (SAFE≤8) was present in 35% [95% CI: 30%-39%] at a median of 1-day post-stroke, with 22% presenting with mild-moderate weakness (SAFE5-8). The most common other impairments were upper limb coordination (46%), delayed recall (41%), and upper limb sensation (26%). After a median 3-day acute stroke stay, 52% of the sample were discharged home.

**Conclusion:**

Upper limb weakness was present in just over a third (35%) of the sample early post-stroke. Data on pre-stroke outcomes and the prevalence of other post-stroke impairments highlights the complexity and heterogeneity of stroke recovery. Further research is required to tease out meaningful recovery phenotypes and their implications.

## Introduction

A stroke occurs every 3 seconds globally, with 1 in 4 people experiencing a stroke in their lifetime.^1^ Acute stroke care has seen the introduction of practice-changing breakthroughs, including thromboloysis^2,3^ and thrombectomy.^4,5^ Understanding the critical time window for acute intervention and the patients likely to respond to treatment has reduced the number of people dying and the overall severity of impairment post-stroke.^6,7^ Considering these changes in acute care, it is necessary to understand the current prevalence of common impairments early post-stroke. This information can help determine the needs of people living with stroke and guide the delivery of rehabilitation services.

Upper limb impairment is common early post-stroke^8-10^ and profoundly impacts quality of life.^11,12^ The Copenhagen stroke study^8^ reported that upper limb motor weakness was present in 69.0% (95% CI: 64%-73%, n = 421 data collected 1992-1993) of acute stroke patients at a median of 13-hours post-stroke.^8^ The Scandinavian Stroke Scale was used to measure upper limb impairment, with a score of ≤5 out of 6 denoting weakness (equivalent to anything below normal strength). In comparison, a more recent study by Simpson et al^9^ reported 40% (95% CI: 36%-44%, n = 621 data collected 2016-2017) of first-ever stroke patients presented with upper limb motor weakness at a median of 24-hours post-stroke. Here, Shoulder Abduction, and Finger Extension (SAFE) score^13,14^ was used to measure upper limb weakness, with a score of ≤8 out of 10 denoting weakness (determined by the sum of Medical Research Council strength whole gradings of shoulder abduction and finger extension).^9^ There was a 30% difference in the percentage of upper limb weakness reported across these 2 studies conducted over 20-year apart.^8-10^ During these 2 decades, thrombolysis^2,3^ and thrombectomy^4,5^ were introduced into routine stroke care. While such interventions are potential explanatory variables, when and how upper limb weakness was measured also requires consideration.^9^

Even fewer studies have examined the prevalence of multiple impairments early-post.^10^ Lawrence et al^10^ extracted registry data of 1259 first-time stroke patients between 1995 and 1998 to determine the prevalence of impairments within 3-weeks post-stroke. Similar to the Copenhagen study, upper limb weakness was present in 77% [95% CI 75%-80%] of the sample. However, there was no description of how upper limb weakness was measured. On average, patients presented with a total of 6.5 (SD 2.95) impairments out of a possible 15, with over half (50.6%) presenting with 3 to 5 impairments.^10^ A cognitive impairment was identified in 44% of the sample as defined by a Mini Mental State Score of 23 or less.^10^ Visual field and inattention impairments were identified in 26% and 20% of the sample via patient confrontation screening.^10^ Upper limb sensory impairment was reported in 30% of the sample through the simultaneous stimulation of both limbs.^10^ This study highlights the prevalence of post-stroke impairments across multiple domains (eg, motor, cognitive, sensory, and visual) and adds to the discussion that stroke recovery is complex.^10^ In a recent paper by the International Stroke Recovery and Rehabilitation Alliance, impairments across multiple domains, the heterogeneity of impairment severity and the patient’s individual circumstances (eg, pre-stroke health, social situation) were some of the factors identified as contributors to the complexity of stroke recovery.^15^ Given the data were collected 25 years ago (prior to the introduction of thromboloysis^2,3^ and thrombectomy^4,5^) by Lawrence et al,^10^ a more contemporary understanding of the prevalence of multiple post-stroke impairments (including upper limb weakness) and the potential impact of pre-stroke health is required.

An interdisciplinary assessment within 24 to 48 hours post-stroke is a practice recommendation within the Australian Clinical Practice Stroke Guidelines.^16^ This assessment, established by each hospital, typically records the presence or absence of impairments (eg, cognitive, visual, perceptual, motor, and sensory) and current functional capacity. It can also record pre-stroke outcomes using measures such as the Modified Rankin Scale (mRS)^17^ and Clinical Frailty Scale (CFS).^18^ The mRS categorizes the level of functional independence with reference to pre-stroke activities. In contrast, the CFS assesses for frailty which is defined as a *“syndrome entailing a state of vulnerability characterized by the cumulative multi-system decline in physiological reserves following a stressor event.”*^19^ The routine administration of measures post-stroke via an interdisciplinary assessment can provide data to benefit clinical and research practice. Clinicians can use this data to (1) advocate for changes to service delivery to meet evolving patient needs, (2) collaborate on discharge needs, goals, and treatment options with the patient, and (3) objectively assess (and re-assess) patient progress. Researchers can use this data to (1) appropriately plan clinical trials by understanding the accessible population available for recruitment,^20^ (2) select priority areas for future research, and (3) understand the range of presenting impairment profile phenotypes and where they end up post-stroke, that is, discharge destination.

The primary aim of this study was to describe the prevalence of upper limb motor weakness (measured using SAFE) early post-stroke at a metropolitan tertiary hospital in Australia. The secondary aim was to contextualize the prevalence of upper limb weakness by describing routinely screened pre-stroke outcomes (eg, mRS and CFS), other post-stroke impairments (eg, cognition, perception, and upper limb sensation/coordination), functional activities (eg, personal activities of daily living and ambulation), and discharge information.

## Method

### Study Design

This cross-sectional observational study was reported in line with the REporting of studies Conducted using Observational Routinely-collected Data^21^ statement. Data were extracted retrospectively from the electronic medical records of a consecutive sample of patients admitted to a metropolitan tertiary hospital under the stroke medical team between 1 April 2021 and 30 July 2022 (15-months). This hospital offers a comprehensive stroke service, including an acute stroke unit, thrombolysis, thrombectomy, and neurosurgery as required. This study obtained ethics approval (HREC/87629) in line with the Declaration of Helsinki.

### Participants

A consecutive sample of admitted patients were reviewed for eligibility. Patients were included if they had a confirmed diagnosis of ischemic or hemorrhagic stroke (not necessarily first stroke) on either inpatient or outpatient imaging (computer tomography [CT] or magnetic resonance imaging [MRI]). Diagnoses of transient ischemic attack, subarachnoid hemorrhage, or cerebral venous thrombosis were excluded from the final dataset, along with patients where it was not appropriate to complete the required clinical assessments (eg, receiving end-of-life care).

### Data Extraction

De-identified data routinely collected by the clinical team were entered into a REDCap database^22^ in line with hospital policy (ED/RJ). A sample of 15% was independently cross-checked for reliability (ED/RJ). For a full list of data points extracted, see Supplemental Table 1. The timing of all data points aligned within the first 24 to 48 hours post-stroke.^16^ It is important to note that data collection was completed during the COVID-19 pandemic and reflects clinical assessment within an Australian hospital.

Demographic (eg, age, gender, premorbid conditions, and social history) and clinical characteristics (eg, stroke type, severity, and acute intervention) were extracted from the interdisciplinary team documentation notes. The clinical measure to assess stroke severity at admission was the National Institutes of Health Stroke Scale (NIHSS),^23,24^ sub-grouped for this study as <5 mild impairment, 5 to 14 mild to moderate impairment, 15 to 24 severe, and >24 very severe.^24^

The SAFE score was the primary measure of upper limb weakness extracted from the usual care neurological assessment developed to meet the practice recommendation in the Australian Clinical Practice Stroke Guidelines.^16^ It was administered by therapists on initial contact with all stroke patients and was anecdotally reported to take between 2 and 5 minutes to complete. The SAFE score is the sum of the Medical Research Council strength whole gradings of 0 to 5 for shoulder abduction and finger extension.^13,14^ The SAFE score was chosen as the upper limb weakness measure because it has shown promise in understanding upper limb recovery trajectory post-stroke^13,14,25,26^ and aligns with recent efforts to understand the prevalence of upper limb weakness early after stroke.^9^ In the current study, we defined upper limb motor weakness as a SAFE score of 0 to 8,^9,14^ which was sub-grouped as severe (SAFE 0-4), mild to moderate (SAFE 5-8), or little to no (SAFE 9-10)^14^ weakness.

Secondary data points extracted were pre-stroke outcomes, other post-stroke impairments, functional activities, and discharge information. All included data points were extracted from the same usual care neurological assessment. Pre-stroke outcomes were assessed via the mRS^17^ and CFS.^18^ The classification of premorbid dependent function was an mRS score of 3 to 5,^27^ and frailty was a CFS score of 5 to 8.^28^ Ten other post-stroke impairments were extracted spanning 6 different impairment domains (cognition, vision, perception, sensory, motor, and complications): command following, delayed recall, visual field deficits, visual tracking, visual inattention/neglect, upper limb coordination, upper limb light touch sensation, upper limb subluxation, upper limb pain, and upper limb tone. The classification of these 10 impairments as intact or impaired was based on individual screening assessments described in Supplemental Table 1. Three functional activities were extracted; sit-to-stand transfers, ambulation, and personal activities of daily living (eg, eating, toileting, dressing, and showering). These activities were rated as independent (ie, safe with or without a gait aid and not requiring the supervision or assistance of another person) or dependent. The other impairments and functional activity data points were pragmatically selected as they were the most reliably reported (>40%) by the clinicians within the usual care neurological assessment. Finally, discharge information, including length of stay (in days) and destination (eg, home and inpatient rehabilitation) were extracted. Therapists received training on the administration method of the primary and secondary outcome measures, with a specific focus on the accurate administration of the SAFE score (primary measure) via written and video materials.

### Statistical Analysis

Descriptive statistics (median [interquartile range, IQR], percentage of the sample [%]) were used to characterize the total sample and the sample by upper limb severity subgroups (defined using the SAFE score). Categorical data were reported as counts and percentages, and continuous data were reported as median [IQR]. The relationship between number of impairments, SAFE score and discharge destination was visually summarized using an alluvial plot. 95% confidence intervals were calculated for the proportion of participants who experienced upper limb weakness. As this study was descriptive in nature, no formal hypothesis testing was performed.

## Results

**Figure 1.**
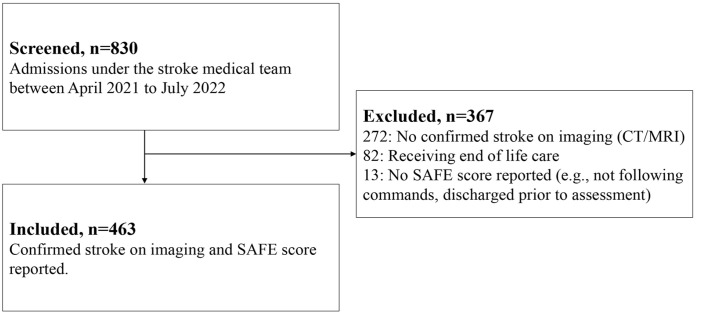
Study flowchart of screened patients between 1 April 2021 and 30 July 2022.

A total of 830 patients were admitted under the stroke medical team over a 15-month screening period, with 463 participants included in the final sample (Figure 1). The most common exclusion reason was no confirmed stroke diagnosis on imaging (74.1%, n = 272), followed by receiving end-of-life care (22.3%, n = 82; Figure 1). Only 13 patients (1.6%) with a confirmed stroke were excluded due to a missing SAFE score (Figure 1). Often, these patients were discharged before assessment or could not follow 1-stage visual or verbal commands to complete the SAFE score (Figure 1).

The demographics and clinical characteristics of the sample (n = 463) are reported in Table 1. The median [IQR] age of the sample was 74.0 [63.0, 83.0], with the majority (58.1%, n = 269) being males. Just over 15% (n = 71) of the sample had experienced a prior stroke, and 6% (n = 29) had a confirmed diagnosis of dementia (Table 1). Most participants had an ischemic stroke (89.6%, n = 415) that was mild in severity as indexed by a median [IQR] admission total NIHSS score of 5.0 [2.0, 10.0]. A third (32.4%, n = 150) of participants received 1 or more acute stroke interventions, with 21.0% (n = 97) receiving thrombolysis and 20.3% (n = 94) receiving thrombectomy.

**Table 1.** Demographics, Clinical Characteristics, and Premorbid Function for the Total Sample and Upper Limb Impairment Groups.

	Sample	Severe	Mild to moderate	Little to no
	SAFE 0-10	SAFE 0-4	SAFE 5-8	SAFE 9-10
Values are n (%) unless otherwise specified	n = 463 (%)	n = 60	n = 101	n = 302
DEMOGRAPHICS				
Age, median [IQR]	74.0 [63.0, 83.0]	72.0 [65.0, 82.3]	79.0 [68.0,85.0]	73.0 [61.0,82.0]
Female	194 (41.9)	22 (36.7)	54 (53.5)	118 (39.1)
Past medical history				
Prior stroke	71 (15.3)	8 (13.3)	17 (16.8)	46 (15.2)
Prior TIA	22 (4.8)	2 (3.3)	4 (4.0)	16 (5.3)
Dementia	29 (6.3)	4 (6.7)	9 (8.9)	16 (5.3)
Other neurological*	28 (6.0)	7 (11.7)	6 (5.9)	15 (5.0)
Upper limb*	88 (19.0)	9 (15.0)	32 (31.7)	47 (15.6)
PRESENTING CLINICAL CHARACTERISTICS				
Stroke side				
Right	237 (51.2)	33 (55.0)	48 (47.5)	156 (51.7)
Left	218 (47.1)	26 (45.0)	50 (49.5)	142 (47.0)
Bilateral	8 (1.7)	0 (0)	3 (3.0)	4 (1.3)
Stroke type				
Ischemic	415 (89.6)	48 (80.0)	95 (94.1)	272 (90.1)
Hemorrhagic	48 (10.4)	12 (20.0)	6 (5.9)	30 (9.9)
Admission NIHSS score, median [IQR]				
Total	5.0 [2.0, 10.0]	7.0 [3.0,12.5]	7.0 [4.0,11.0]	4.0 [2.0,8.6]
Upper limb subscale	1.0 [0.0, 2.0]	1.0 [0,3.0]	1.0 [0,2.0]	0 [0,1.0]
Acute intervention				
Received ≥1 intervention	150 (32.4)	35 (58.3)	36 (35.6)	79 (26.2)
Thrombectomy	94 (20.3)	25 (41.7)	26 (25.7)	43 (14.2)
Thrombolysis	97 (21.0)	14 (23.3)	25 (24.8)	58 (19.2)
Neurosurgery	14 (3.0)	6 (10.0)	0 (0)	8 (2.6)
Acute length of stay in days, median [IQR]	3.0 [2.0, 7.0]	9.5 [6.0, 20.3]	4.0 [2.0,8.0]	2.0 [1.0,4.8]
Discharge location*				
Home	240 (51.8)	0 (0)	33 (32.7)	207 (68.5)
Acute transfer	35 (7.7)	14 (23.3)	12 (11.9)	9 (3.0)
Rehabilitation at home	19 (4.1)	4 (6.7)	4 (4.0)	11 (3.6)
Fast stream rehab	109 (23.5)	23 (38.3)	28 (27.7)	58 (19.2)
Public	93 (85.3)	22 (95.7)	24 (85.7)	47 (81.0)
Private	16 (14.7)	1 (4.3)	4 (14.3)	11 (19.0)
Slow stream rehab	45 (9.7)	15 (25.0)	19 (18.8)	11 (3.7)
Residential care	15 (3.2)	4 (6.7)	5 (4.9)	6 (2.0)
PREMORBID FUNCTION				
Living situation				
Home alone	102 (22.0)	14 (23.3)	24 (23.8)	64 (21.2)
Home with someone	305 (65.9)	37 (61.7)	62 (61.4)	206 (68.2)
RACF	24 (5.2)	7 (11.7)	7 (6.9)	10 (3.3)
Not reported	32 (6.9)	2 (3.3)	8 (7.9)	22 (7.3)
Formal services*				
Yes	68 (14.7)	13 (21.7)	14 (13.9)	41 (13.6)
Not reported	94 (20.3)	11 (18.3)	18 (17.8)	65 (21.5)
Hand dominance				
Right-handed	365 (78.8)	48 (80.0)	78 (77.2)	239 (79.1)
Not reported	58 (12.5)	6 (10.0)	14 (13.9)	38 (12.6)
CFS, score between 1 and 9				
Not Frail (CFS 1-4)	331 (71.5)	33 (55.0)	61 (60.4)	237 (78.5)
Not reported	30 (6.5)	7 (11.7)	5 (5.0)	18 (6.0)
Median total score [IQR]	3.0 [2.0,5.0]	3.0 [2.0, 6.0]	3.0 [2.0,5.0]	3.0 [2.0,4.0]
mRS, score between 0 and 6				
Independent (mRS 0-2)	333 (71.9)	36 (60.0)	65 (64.4)	232 (76.8)
Not reported	26 (6.5)	6 (10.0)	2 (2.0)	18 (6.0)
Median total score [IQR]	0.0 [0.0,3.0]	1.0 [0,3.0]	1.0 [0,3.0]	0 [0,2.0]
Ambulation				
Independent	425 (91.8)	48 (80.0)	92 (91.1)	285 (94.4)
Assistance	18 (3.9)	5 (8.3)	6 (5.9)	7 (2.3)
Not reported	20 (4.3)	7 (11.7)	3 (3.0)	10 (3.3)
Mobility aid				
Yes	71 (15.3)	12 (20.0)	18 (17.8)	41 (13.6)
Not reported	19 (4.1)	5 (8.3)	1 (1.0)	13 (4.3)
Falls history				
Yes	53 (11.4)	3 (5.0)	10 (10.0)	40 (13.3)
Not reported	19 (4.1)	6 (10.0)	6 (5.9)	20 (6.6)
Personal ADLs				
Independent	382 (82.5)	36 (60.0)	80 (79.2)	266 (88.1)
Assistance	62 (13.4)	16 (26.7)	20 (19.8)	26 (8.6)
Not reported	19 (4.1)	8 (13.3)	1 (1.0)	10 (3.3)
Domestic ADLs				
Independent	269 (58.1)	27 (45.0)	49 (48.5)	193 (63.9)
Assistance	175 (37.8)	25 (41.7)	51 (50.5)	99 (32.8)
Not reported	19 (4.1)	8 (13.3)	1 (1.0)	10 (3.3)
Community ADLs				
Independent	274 (59.2)	27 (45.0)	50 (49.5)	197 (65.2)
Assistance	165 (35.6)	23 (38.3)	49 (48.5)	93 (30.8)
Not reported	24 (5.2)	10 (16.7)	2 (2.0)	12 (4.0)

Abbreviations: ADL, activities of daily living; CFS, Clinical Frailty Scale; mRS, modified Rankin Scale; NIHSS, National Institutes of Health Stroke Scale; RACF, residential age care facility.

* *Other neurological* includes Parkinson’s Disease, Multiple Sclerosis, Vertigo, Acquired Brain Injury, Epilepsy, Intellectual Disability, and Subarachnoid hemorrhage. *Upper limb included any premorbid condition that impacted the use of the impaired upper limb before the stroke (eg, arthritis and rotator cuff injury). *Formal services are paid services that patients receive in the community (eg, cleaning). *Discharge location: *Home* refers to discharge to a community-dwelling. *Acute transfer* refers to the participants who were transferred to another acute hospital that is usually closer to their residential address, or they were admitted there first and then transferred to our hospital for specialized stroke care (eg, thrombectomy). *Rehabilitation at home* refers to a short-term, multidisciplinary rehabilitation program provided to patients within their own home. *Fast-stream rehabilitation* refers to multidisciplinary inpatient rehabilitation in Australia, typically provided at a higher intensity therapy and was either publicly funded under Medicare or privately funded by a patient’s private health insurance. *Slow-stream rehabilitation*, commonly referred to as geriatric evaluation and management in Australia, typically provided less intensive therapy and was more targeted to patients over the age of 65 (publicly funded service). *Residential care* refers to an aged care home, patients may have been a resident of this care facility on admission to the acute stroke unit or may have been a new admission after their stroke.

**Figure 2.**
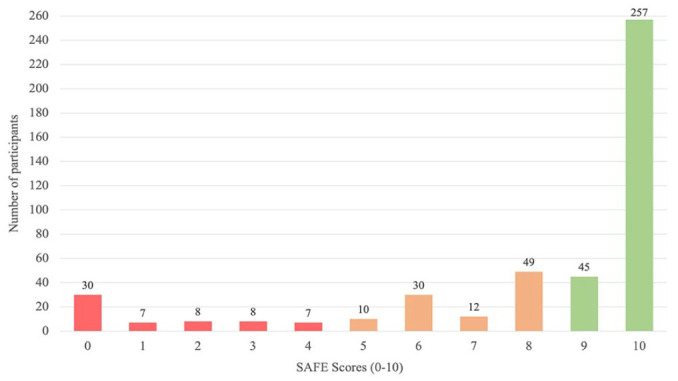
Prevalence of upper limb motor weakness early post-stroke classified by severity subgroup. Abbreviation: SAFE score, Shoulder Abduction and Finger Extension Score. *Note*. SAFE severity subgroups classified based on prior research.^14^ Little to no refers to a SAFE of 9 and 10 (green). Mild to moderate refers to a SAFE of 5 to 8 (orange). Severe refers to a SAFE of 0 to 4 (red).

The SAFE score was administered at a median [IQR] of 1.0 [1.0,2.0] day post-stroke. Over a third (34.8% [95% CI: 30.4%-39.1%], n = 161) of the sample had upper limb motor weakness (SAFE 0-8; see Figure 2). The percentage of upper limb motor weakness increased to 44.5% (n = 206) when a SAFE range of 0 to 9 was applied. Thirteen percent (n = 60) presented with severe weakness (SAFE 0-4), 21.8% (n = 101) presented with mild to moderate weakness (SAFE 5-8), and 65.2% (n = 302) presented with little to no weakness (SAFE 9-10); see Figure 2.

Premorbid, most of the sample lived with someone (65.9%, n = 305), were independently mobile (91.8%, n = 425), and independent in personal activities of daily living (82.5%, n = 382), Table 1. The median mRS score was 0.0 [0.0, 3.0] and the median [IQR] CFS score was 3.0 [1.0, 4.0], indicating that most participants had “*no disability*” and were “*managing well*” before admission (Table 1). Twenty-two percent (n = 101) of the sample were classified as frail (CFS 5-9) prior to their stroke admission. The percentage of frail participants was higher in the severe (33.3%, n = 20) and mild to moderate (34.7%, n = 35) upper limb severity subgroups but lower in the little to no upper limb severity subgroup (15.6%, n = 47). Of those participants who were not frail (CFS 1-4), 58.0% (n = 192) were discharged directly home from the acute stroke unit, while only 33.3% (n = 34) classified as frail (CFS 5-9) went directly home.

Other impairments and functional activities were assessed at the same timepoint post-stroke as the SAFE score (median [IQR] of 1.0 [1.0,2.0] days post-stroke) and are reported in Table 2. At this early timepoint, only a small percentage of the sample were assessed as impaired with command following (single stage, 6.6%, n = 25), upper limb subluxation (1.5%, n = 6), upper limb pain (2.6%, n = 8), and upper limb tone (7.4%, n = 28). Upper limb coordination deficits were present in 45.5% (n = 152) of the sample, with the mild to moderate subgroup representing the highest percentage of impaired participants (76.8%, n = 47). Upper limb sensory deficits were present in a quarter of the sample (25.5%, n = 94), with the severe subgroup being the highest percentage of impaired participants (71.2%, n = 28). Nearly 60% (n = 254) of the sample could independently sit to stand, but less than half were independently ambulant (45.9%, n = 199) or independent with personal activities of daily living (40.5%, n = 166). No participants in the severe upper limb subgroup were independent with personal activities of daily living.

**Table 2.** Prevalence of Other Post-stroke Impairments and Functional Activities in the Sample and Upper Limb Severity Subgroups.

Other impairment or functional activity*	Number assessed (%)	Number impaired or dependent (%)
COGNITIVE DOMAIN		
Command following		
Sample^a^	381 (82.3)	25 (6.6)
Severe^b^	43 (71.7)	12 (27.9)
Mild to moderate^c^	93 (57.8)	6 (6.5)
Little to no^d^	245 (81.1)	7 (2.9)
Delayed recall		
Sample^a^	222 (47.9)	92 (41.4)
Severe^b^	15 (25.0)	10 (66.7)
Mild to moderate^c^	53 (52.5)	29 (54.7)
Little to no^d^	154 (51.0)	53 (34.4)
VISUAL DOMAIN		
Visual field deficits		
Sample^a^	336 (42.6)	47 (14.0)
Severe^b^	33 (55.0)	5 (15.2)
Mild to moderate^c^	72 (71.3)	9 (12.5)
Little to no^d^	231 (76.5)	33 (14.3)
Visual tracking		
Sample^a^	338 (73.0)	52 (15.4)
Severe^b^	33 (55.0)	9 (27.3)
Mild to moderate^c^	67 (66.3)	11 (16.4)
Little to no^d^	238 (78.8)	32 (13.4)
PERCEPTUAL DOMAIN		
Visual inattention/neglect		
Sample^a^	337 (72.8)	48 (14.2)
Severe^b^	36 (60.0)	10 (27.8)
Mild to moderate^c^	70 (69.3)	15 (21.4)
Little to no^d^	231 (76.5)	23 (10.0)
SENSORY DOMAIN		
Upper limb light touch sensation		
Sample^a^	369 (79.7)	94 (25.5)
Severe^b^	39 (65.0)	28 (71.2)
Mild to moderate^c^	75 (74.3)	32 (42.7)
Little to no^d^	255 (84.4)	34 (13.3)
MOTOR DOMAIN		
Upper limb coordination*		
Sample^a^	334 (72.1)	152 (45.5)
Severe^b^	13 (21.7)	9 (69.2)
Mild to moderate^c^	63 (62.4)	47 (74.6)
Little to no^d^	258 (85.4)	96 (37.2)
COMPLICATIONS DOMAIN		
Shoulder subluxation		
Sample^a^	390 (84.2)	6 (1.5)
Severe^b^	39 (65.0)	4 (10.3)
Mild to moderate^c^	90 (89.1)	0 (0)
Little to no^d^	261 (86.4)	2 (0.8)
Upper limb pain		
Sample^a^	312 (67.4)	8 (2.6)
Severe^b^	31 (51.7)	3 (9.7)
Mild to moderate^c^	70 (69.3)	4 (5.7)
Little to no^d^	211 (69.9)	1 (0.5)
Upper limb tone		
Sample^a^	378 (81.7)	28 (7.4)
Severe^b^	40 (66.7)	18 (45.0)
Mild to moderate^c^	81 (80.2)	9 (11.1)
Little to no^d^	257 (85.1)	1 (0.4)
FUNCTIONAL ACTIVITIES		
Sit to stand transfers		
Sample^a^	426 (92.0)	172 (40.4)
Severe^b^	45 (75.0)	44 (97.8)
Mild to moderate^c^	96 (95.0)	53 (55.2)
Little to no^d^	285 (94.4)	75 (26.3)
Ambulation		
Sample^a^	434 (93.7)	235 (54.1)
Severe^b^	50 (83.3)	49 (98.0)
Mild to moderate^c^	95 (94.1)	65 (68.4)
Little to no^d^	287 (95.0)	119 (41.5)
Personal ADLs		
Sample^a^	410 (88.6)	244 (59.5)
Severe^b^	43 (71.7)	43 (100.0)
Mild to moderate^c^	90 (89.1)	71 (78.9)
Little to no^d^	277 (91.7)	130 (46.9)

Abbreviation: ADLs, activities of daily living.

a Sample includes 463 participants.

b Severe includes 60 participants.

c Mild to moderate includes 101 participants.

d Little to no includes 302 participants.

* Impairment/activity: a full description of how these impairments were assessed can be found in Supplemental Table 1. *Upper limb coordination: note that patients who were unable to participate in the assessment due to motor weakness were considered intact.

**Figure 3.**
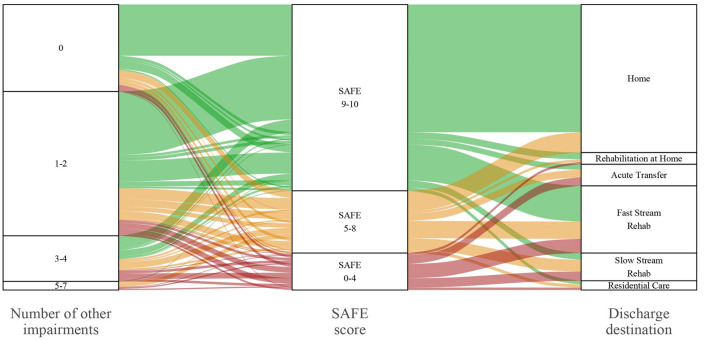
Alluvial plot representing upper limb severity subgroups (middle column), number of other post-stroke impairments (left-hand column), and discharge destination from the acute stroke unit (right-hand column). Abbreviation: SAFE, Shoulder Abduction and Finger Extension Score. *Note*. The alluvial plot originates from the middle column (primary outcome, SAFE scores) and continues to the left-hand column (number of other impairments) and the right-hand column (discharge destination). *Number of other post-stroke impairments* refers to the number of additional impairments (out of 10) that patients presented with (see Supplemental Table 1). *Discharge location* refers to where participants were discharged after their acute stroke unit stay. *Home* refers to discharge to a community-dwelling. *Rehabilitation at home* refers to a short-term, multidisciplinary rehabilitation program provided to patients within their own home. *Acute transfer* refers to the participants who were transferred to another acute hospital that is usually closer to their residential address, or they were admitted there first and then transferred to our hospital for specialized stroke care (eg, thrombectomy). *Fast-stream rehabilitation* refers to multidisciplinary inpatient rehabilitation, often delivered at a higher intensity than slow-stream rehabilitation. *Slow-stream rehabilitation* is usually only offered to patients over the age of 65. *Residential care* refers to an aged care home, patients may have been a resident of this care facility on admission to the acute stroke unit or may have been a new admission after their stroke.

To further contextualize the findings, Figure 3 visually represents the interaction between the upper limb severity subgroups (primary outcome), other post-stroke impairments (secondary outcome), and discharge location (secondary outcome). Of participants that had upper limb weakness (SAFE 0-8, n = 161), 21.8% (n = 35) presented with no other impairments, 30.4% (n = 49) presented with 1, 17.4% (n = 28) presented with 2, 11.8% (n = 19) presented with 3, 10.6% (n = 17) presented with 4 and 8.0% (n = 13) presented with 5 to 7 other impairments. No participants had 8 or more other impairments (see Supplemental Table 2). Of the total sample (SAFE 0-10, n = 463), 40.9% (n = 190) presented with impairments across 2 or more domains (eg, motor and vision). Around half (51.8%, n = 240) of the sample went directly home from the acute stroke unit, and 33.2% (n = 154) of the sample went to either fast or slow-stream rehabilitation (see Table 1 and Figure 3). No participants in the severe upper limb subgroup went directly home. The median length of stay in the acute stroke unit was 3.0 days [2.0,7.0] (Table 1). Length of stay increased with upper limb severity. Participants in the severe upper limb subgroup (median 9.5 days) stayed over 4 times longer than those in the little to no upper limb subgroup (median 2.0 days; Table 1). Interestingly, 78.1% of those with no impairments were discharged home compared to 44.1% of those with 1 more impairment.

## Discussion

This observational study of clinical data from a tertiary acute stroke unit in Australia found that nearly 35% [95% CI: 30%-39%] of people experienced upper limb weakness at a median of 1-day post-stroke. A greater percentage of the sample had mild to moderate weakness than severe weakness. Across all participants, the 3 most common other post-stroke impairments (out of 10 screened) were upper limb coordination, delayed recall, and upper limb light touch sensation. After a median 3-day stay on the acute stroke unit, just over half of the sample went home, but this did not include any participants in the severe upper limb subgroup. A higher percentage of people presenting with no premorbid frailty (58.0%) or no other impairments (78.1%) went home, which is in contrast to people presenting with premorbid frailty (33.3%) or 1 or more impairments (44.1%). Our contextualization of the prevalence of upper limb weakness with pre-stroke outcomes and other post-stroke impairments has demonstrated the complex and heterogenous presentation of people early after stroke.

**Figure 4.**
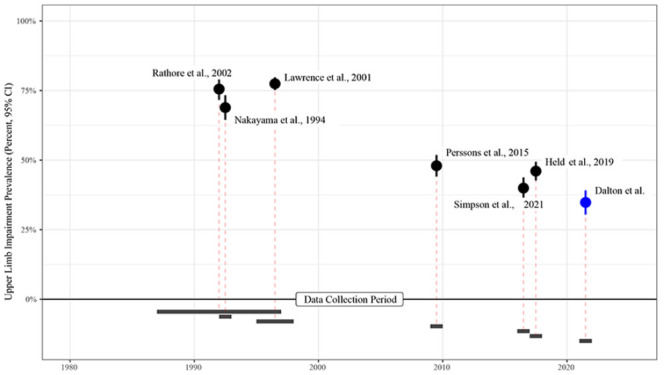
Percentage (95% CI) of upper limb weakness in published trials^8-10,29,30,33^ and current study sample (Dalton et al) based on year(s) data collection.

The percentage (95% CI) of upper limb motor weakness in this study was consistent with data recently collected at a tertiary acute stroke unit in Canada (Australia: 35.0% 95% CI: 30%-39% and Canada: 40.0% 95% CI: 36%-44%). The Canadian site also administered the SAFE score at a consistent time point post-stroke (median 1-day).^9^ Both sites also had a similar percentage of participants with an ischemic stroke (Australia: 89.6% and Canada: 87.4%) and who received an acute stroke intervention (Australia: 32.4% and Canada: 32.0%).^9^ This result prompted the consideration of whether there has been a shift over time in the number of people presenting with upper limb motor weakness early post-stroke (see Figure 4). Interestingly, there appear to be 2 distinct groups of studies based on data collection period. The earlier group (data collected between 1987 and 1998) reported a percentage of upper limb weakness that ranged from 69% to 77%, while the later cluster (data collected between 2009 and 2022) reported a percentage of upper limb weakness that ranged from 35% to 48%. There were no studies in the earlier cluster that reported the use of thrombolysis and thrombectomy as it was not part of routine stroke care at this time. All studies^9,29,30^ in the later cluster reported the use of thrombolysis and thrombectomy (range between 9.9% and 32.0% of the included sample). These acute interventions,^2-5^ along with a stronger focus on primary stroke prevention^31^ and improved imaging techniques to identify small strokes^32^ are some possible explanations that may have contributed to the observed differences over time. However, further research is warranted to understand whether these advances in stroke care or other explanations have driven the decline in upper limb weakness prevalence highlighted in Figure 4. It is important to acknowledge when reviewing Figure 4, different upper limb measures were used to determine the percentage (95% CI) of weakness across the included studies. It will be interesting to see if recent prevalence findings continue to be observed in high-income centers (rural, regional, and metropolitan) and to understand what is occurring in low- to middle-income countries.

It was challenging to draw comparisons regarding any potential shifts in the percentage of other post-stroke impairments as studies of upper limb weakness infrequently measure other impairments and use different outcomes at different time points post-stroke. Only visual field deficits, visual inattention and upper limb sensory impairments were assessed similarly in this study (data collected 2021-2022) and the study by Lawrence et al^10^ (data collected 1995-1998). The percentage of all 3 impairments was over 5% lower in the current study than observed in the earlier study.^10^ These findings need to be interpreted with caution given the assessment time point varied between the 2 studies (median 1-day post-stroke vs within 3-weeks of stroke^10^). Additional research is required to confirm the lower percentage of visual field, visual inattention, and upper limb sensory impairment reported in this study as a decline that is generalizable across stroke recovery. Consistent with upper limb weakness, further investigation of the possible explanations for the observed differences (eg, advances in primary stroke prevention^31^ and acute stroke care^2-5,32^) is required.

Reporting how and when post-stroke impairments are measured is necessary to contextualize the data. The consistent administration of the SAFE score in the recent Australian and Canadian^9^ studies has allowed a comparison to be drawn. The field must be consistent in how data is collected to continue to build our understanding of the prevalence of upper limb motor weakness and other impairments early post-stroke. There are trade-offs that must be considered when selecting an outcome. The SAFE score is a simple and efficient measure of upper limb weakness.^13,14^ The small amount of missing data (1.6%) in this study indicated that it was feasible and sustainable for clinicians to administer in a clinical setting, which coincided with pandemic-related lockdowns and associated hospital and staffing impacts. The first Stroke Recovery and Rehabilitation Roundtable recommended (by consensus) the Fugl-Meyer Assessment^34^ as the measure of upper limb motor weakness in stroke recovery trials. This measure provides more granular detail than a SAFE score^13,14^ but is reported to take 20 minutes to complete.^35^ The priorities of outcome measurement in a clinical setting differ from those in a research trial. The median acute length of stay was 3-day in this study, with most participants returning home. Rehabilitation goals and discharge planning are prioritized to ensure safe discharge. Using an efficient measure (such as the SAFE score) on the acute stroke unit has allowed the administration of a standardized upper limb outcome to become part of usual care within the current length of stay constraints.

The observed prevalence of upper limb motor weakness has implications for service delivery. Nearly 70% of people with little to no upper limb weakness (SAFE 9-10) were discharged directly home at a median of 2-day post-stroke. The treating team deemed these participants functionally safe for discharge, but they often had ongoing difficulties using their impaired upper limb that would warrant a referral to community rehabilitation. Earlier work by Stewart and Cramer^36^ also demonstrated that participants scoring well on an impairment-based upper limb measure (ie, Fugl-Meyer) still report ongoing upper limb difficulties. The wait time for community rehabilitation in Australia is highly variable and dependent on where the person after stroke lives.^37^ Often there is a gap of months between discharge from the acute stroke unit and the commencement of therapy. This is of concern, as people after stroke and their families often report the early period after discharge as the most difficult time.^37^ Further consideration is needed about whether the current service model remains fit for purpose. The rise of telehealth and platforms to support self-managed exercise programs at home during the COVID-19 pandemic could be one solution to address this service gap.^38^ Results from this study suggest there remains a vital role for inpatient rehabilitation services, with 46.5% of those with mild to moderate weakness and 63.6% of those with severe weakness discharged to inpatient rehabilitation after stroke. Optimization of inpatient rehabilitation services, especially for people with severe weakness, requires ongoing consideration.

While the prevalence of upper limb weakness and other impairments early post-stroke may be shifting, the complexity of stroke recovery remains an important topic for the stroke recovery community.^15^ The presence of impairments across multiple domains and individual patient circumstances, including premorbid conditions such as dementia and clinical frailty, have implications for researchers and clinicians. Forty-one percent of the participants in this study had impairments across 2 or more domains. This prevalence may be higher if other impairments within the language domain, for example, were considered. Regarding premorbid conditions, a review of the eligibility criteria of upper limb trials conducted 14 days post-stroke determined that 70.0% included a criterion excluding participants with premorbid conditions.^20^ As the population ages, the incidence of dementia and other conditions is predicated to rise.^39^ Including (or not) people with these conditions in stroke recovery trials requires researchers to consider the balance between internal and external validity.^20^ There is also an emerging body of research that suggests premorbid clinical frailty (measured via the CFS) is independently associated with 28-day mortality after ischemic stroke and attenuated improvement in NIHSS post thrombolysis.^28^ The presence of premorbid conditions (including dementia and clinical frailty) is important to understand the complex and heterogeneous nature of stroke recovery. Further research is required to tease apart biologically relevant and clinically meaningful recovery phenotypes to guide research and inform clinical care. Harnessing stroke recovery biomarkers^40^ (eg, motor specific such as corticospinal tract integrity, and whole brain health, such as predicted brain age) with discriminative or predictive capacity and data collected from multiple international sites could also maximize our ability to target the right patients in future research trials and usual care interventions.

## Limitations

Data were collected from a single metropolitan tertiary stroke unit in Australia. Administering the SAFE score at a consistent time point allowed comparisons with another international single-site sample.^9^ The generalizability of these findings could be improved by including more sites, including regional sites within Australia where the percentage of acute interventions may be less, and collaboration with other international sites to confirm the global upper limb motor weakness prevalence. Using routinely collected data meant we could not compare the NIHSS upper limb subscale and SAFE scores as they were administered at different time points in routine clinical care (NIHSS at admission and SAFE score at a median 1-day post-stroke). There were also no routinely collected upper limb activity outcome measures, post-stroke functional outcome measures, or contextualizing discharge information such as the presence of a primary carer available, which could strengthen the discussion in this study. This study included a single assessment timepoint, with no longitudinal follow-up of participants to track recovery, which could be addressed in future studies. The additional post-stroke impairments assessed were pragmatically selected from the usual care assessment tool based on the consistency of reporting. For example, only light touch sensation was consistently reported and therefore included. The overall number of patients with sensory impairment could differ from the reported 26% if other sensory impairments were included (eg, temperature). The clinical tool did not assess communication impairments (eg, aphasia). Therefore, no data were available on this impairment domain which is known to impact recovery post-stroke. Future observational studies should look to include communication-related impairment outcomes.

## Conclusion

This cross-sectional observational study observed a lower percentage of patients presenting with upper limb motor weakness than well-cited studies from over 30 years ago.^8-10^ This result was consistent with recent research using the SAFE score at a consistent time point post-stroke.^9^ The contextualization of upper limb weakness with both pre-stroke and other post-stroke impairments highlights the complex and heterogenous presentation of people early after stroke. Further research is required to tease out meaningful phenotypes early post-stroke and their implications for recovery, service delivery, and clinical trial sampling and recruitment.

## Supplemental Material

sj-docx-1-nnr-10.1177_15459683241229676 – Supplemental material for Prevalence of Arm Weakness, Pre-Stroke Outcomes and Other Post-Stroke Impairments Using Routinely Collected Clinical Data on an Acute Stroke Unit Supplemental material, sj-docx-1-nnr-10.1177_15459683241229676 for Prevalence of Arm Weakness, Pre-Stroke Outcomes and Other Post-Stroke Impairments Using Routinely Collected Clinical Data on an Acute Stroke Unit by Emily J. Dalton, Rebecca Jamwal, Lia Augoustakis, Emma Hill, Hannah Johns, Vincent Thijs and Kathryn S. Hayward in Neurorehabilitation and Neural Repair
