# Supplementary material for: Prevalence of Arm Weakness, Pre-Stroke Outcomes and Other Post-Stroke Impairments Using Routinely Collected Clinical Data on an Acute Stroke Unit
Source: Neurorehabil Neural Repair. 2024 Feb 10;38(2):148–60. doi: 10.1177/15459683241229676 (PMC10878009; doi:10.1177/15459683241229676)
Supplement: sj-docx-1-nnr-10.1177_15459683241229676 – Supplemental material for Prevalence of Arm Weakness, Pre-Stroke Outcomes and Other Post-Stroke Impairments Using Routinely Collected Clinical Data on an Acute Stroke Unit [file sj-docx-1-nnr-10.1177_15459683241229676.docx]

**SUPPLEMENTAL MATERIALS**

**Title:** Prevalence of arm weakness, pre-stroke outcomes and other post-stroke impairments using routinely collected clinical data on an acute stroke unit.

**Short title:** Arm weakness early post-stroke.

**Corresponding author:**

A/Prof Kate Hayward, PhD

Department of Physiotherapy, University of Melbourne, Australia.

University of Melbourne. Level 7 Alan Gilbert Building, 161 Barry Street Carlton, VIC, AUS

[kate.hayward@unimelb.edu.au](mailto:kate.hayward@unimelb.edu.au)

**Supplemental Material 1:** Data extraction points from usual care assessment.

| **Area** | **Data extracted** |
| --- | --- |
| **Demographics** | - **Age,** years at stroke onset - **Sex,** male or female - **Premorbid conditions:** Prior stroke / prior TIA / dementia / other neurological* / upper limb* |
| **Clinical characteristics** | - **Stroke side:** Left / right / bilateral - **Stroke type:** Ischaemic/ haemorrhagic/ both - **Stroke severity:** Admission NIHSS total score / admission NIHSS upper limb subscale - **Acute stroke intervention:** None / thrombolysis / thrombectomy / neurosurgery / combination - **Acute length of stay (days)** - **Discharge location:** Home / acute transfer / rehabilitation at home / fast stream rehabilitation (private vs public), slow stream rehabilitation / RACF |
| **Premorbid function** | - **Living situation:** Home alone / home with someone / residual aged care facility - **Formal services*:** Yes / no - **Hand dominance:** Left / right / both - **Clinical Frailty Scale (CFS)^17^:** 1: very fit, 2: well, 3: managing well, 4: vulnerable, 5: mildly frail, 6: moderately frail, 7: severely frail, 8: very severely frail and 9: terminally ill. *Classification: 0 to 4 = not frail. 5 to 9 = frail^27^* - **modified Rankin Scale (mRS) score^16^:** 0: no symptoms, 1: no significant disability, 2: slight disability, 3: moderate disability, 4; moderately severe disability, 5: severe disability, 6: death. *Classification: 0 to 2 = independent function. 2 to 5 = not independent function^26^.* - **Ambulation:** Independent / assistance (includes supervision and all levels of assistance) - **Mobility aid:** Yes / no - **Falls history (in last 12 months):** Yes / no - **Personal activities of daily living:** Independent / assistance (includes supervision and all levels of assistance) - **Domestic activities of daily living:** Independent / assistance (includes supervision and all levels of assistance) - **Community activities of daily living:** Independent / assistance (includes supervision and all levels of assistance) |
| **Upper limb motor impairment** | - **Timepoint of assessment post-stroke** (days) - **SAFE** total score /10. - **SAFE subscores**, shoulder abduction score /5, finger extension score /5   *Manual Muscle Testing Method:*  *0 Zero: Has no palpable or visible muscle contraction,*  *1 Trace: Has palpable muscle contraction but no visible movement,*  *2 Poor: Moves joint through full active ROM in a gravity-eliminated position,*  *3 Fair: Completes active ROM against gravity without manual resistance,*  *4 Good: Completes active ROM against gravity; able to hold position against moderate resistance,*  *5 Normal: Completes active ROM against gravity; able to hold position against maximal resistance.* |
| **10 additional post-stroke impairments** | 1. **Command following:** impaired if unable to follow 1x stage commands   *Method: Ask the patient to complete 1, then 2, then 3 stage commands.*  *Observe: Ability to follow 1, 2, 3 stage commands. Consider factors impeding comprehension e.g. receptive dysphasia/loss of hearing*   1. **Delayed recall:** impaired if unable to recall three words after 5 minutes   *Method: After a minimum of a 5-minute delay, ask the patient to state the three words that they repeated earlier.*  *Observe: Document any discrepancies or errors.*   1. **Visual field deficits:** impaired if any deficits found on confrontational assessment   *Method: Cover eye on the non-affected side first. Therapist sits in front of patient. Present a bright object (approx. 30cm away) to patient’s peripheral vision of uncovered eye, slowly bringing it in centrally. Test middle (eye level), upper (forehead level) and lower (chin level) aspects of visual field. Repeat with affected eye covered.*  *Instructions: Ask patient to fixate on central target - “Look at my nose and say stop when you first see the object in the corner of your vision”.*  *Observe: Any discrepancy between quadrants and L/R eyes. Do you suspect visual field loss? Where? Hemianopia is the loss of half of the visual field in one eye. Homonymous hemianopia is the loss of half of the visual field in both eyes. Superior/inferior quadrantanopia is the loss of upper or lower quarter of the visual field.*   1. **Visual tracking:** impaired if any deficits are found on tracking assessment   *Method: Sit in front of the patient; the patient is to look straight ahead at the therapist with his/her head in the midline. Therapist moves pen at even speed about 20cm in front of the patient in horizontal, vertical and diagonal movements.*  *Instructions: “Keep your head still and follow the pen with your eyes as it moves.”*  *Observe: The eyes should move together. Observations are made regarding speed, coordination and ROM of the eyes and ability to cross the midline. Impaired tracking: patient loses focus on the object; deviation of the eyes to (R) or (L) and not crossing midline; impaired ability to focus in superior / inferior quadrants; or unable to look upward (i.e. poor upward gaze)*   1. **Visual inattention / neglect:** impaired if deficits found on extinction assessment   *Method: Therapist to hold index fingers of both hands in patient’s L) and R) peripheral fields. Wiggle one finger at a time, then both together. Check upper and lower quadrants, and check for consistency.*  *Instructions: “Tell me (or point to) which finger you see moving – R) or L) or both”*  *Observe: Visual inattention to ‘X’ (R/L) side, if consistently not able to notice when provided with bilateral input, but can notice/see stimuli in isolation. Always need to test visual field first, as patients with a visual field loss (i.e. hemianopia) will still fail to identify stimuli on the affected side. Patients with inattention will fail to track into the affected side on the visual tracking task.*   1. **Upper limb coordination:** impaired if deficits noted on finger-nose assessment   *Method: Test bilaterally (if able), if impaired make comments on severity: (e.g. mild, severe). Consider impact of sensation / motor skills on coordination.*  *Therapist holds their index finger directly in front of the patient at nose level (finger is at a distance that requires patient to extend their elbow to reach the target).*  *Instructions: Ask the patient to move their index finger rapidly and accurately between their nose and the therapist’s finger. Prevent elbow stabilisation*  *Observe: Dysmetria (under or overshooting target), dyssynergia (wavering to touch at end-point), tremor, bradykinesia (slowed movement), and fatigue*   1. **Upper limb light touch sensation:** impaired if deficits noted on screening assessment   *Method: Ensure you vary the timing and placement of the stimuli to check for accuracy of responses. Test unaffected side first to ensure the patient understands.*  *Instructions: Ask the patient to close their eyes and respond (yes or point to) when they feel the stimulus. Brush cotton (fingertip, tissue etc.) on small areas (3cm of patient skin. Ensure you randomly brush the patient’s right arm only, left arm only, and at times ensure you also brush both arms simultaneously with bilateral stimulation (to assess for sensory inattention/neglect). Ask patient if stimulus is the same or different to unaffected side.*   1. **Upper limb subluxation:** impaired if present on palpation   *Method: This should be assessed prior to passive ranging of the hemiplegic arm. Assessment involves a rough estimation (e.g. cm) of the distance separating the acromial angle and the lateral epicondyle of the humerus. Best assessed in sitting, where possible: place hands on patient’s shoulders. Move hands laterally along bony surface of the shoulder, approximately 5cm down the arm. If subluxation present, index finger will move into a prominent gap between the humeral head and the acromion process. Compare with the unaffected limb.*  *Instructions: Comment on risk of subluxation (i.e. flaccid, low tone, strength <3/5, inattention/neglect = patient likely to be at high risk).*   1. **Upper limb pain:** impaired if present on Ritchie Articulate Index   *Method: Shoulder pain is assessed using a modified Richie Articular Index.*  *Patient lies supine with body supported. Hemiplegic shoulder is abducted to 30 degrees and elbow at 90 degrees of flexion, forearm in neutral. The response and behaviour to this movement are rated.*  *Observe: 3 -point scale. 1- no tenderness, 2- patient complained of pain , 3- patient complained of pain, winced and withdrew.*   1. **Upper limb tone:** impaired if hypotonicity or hypertonicity present on screening assessment   *Method: Assess muscle resistance to slow passive ROM throughout available ROM.*  *Observe: If abnormal, comment generally on the presence of either hyper- or hypo-tonicity. Hypotonicity (flaccid – low tone) –  used to describe muscles that are floppy. On assessment the muscle feels heavy, offers no resistance to PROM, the muscle belly is soft to palpate, usually no reflexive movement, which is also known as hypotonia. Hypertonicity (increased tone) – The limb will feel stiff and difficult to move, there is resistance to PROM when the limb is moved.* |
| **Current function** | 1. **Sit to stand transfers:** impaired if not independent (assessed by physiotherapist). 2. **Ambulation:** impaired if not independent with or without a gait aid (assessed by physiotherapist). 3. **Personal activities of daily living:** impaired if not independent with feeding, toileting, dressing and shower with or without equipment (assessed by occupational therapist) |

**Table 2:** Percentage of the total sample and the upper limb weakness severity subgroups assessed as impaired on each of the possible impairments.

| **Number of impairments** | Percentage of the total sample (SAFE 0-10, n=463) that was assessed as impaired on each of the 11 possible impairments. | Percentage of those with upper limb motor weakness (SAFE 0-8, n=161) that was assessed as impaired on each of the 10 possible impairments. | Percentage of the severe upper limb severity subgroup (SAFE 0-4, n=60) that was assessed as impaired on each of the 10 possible impairments. | Percentage of the mild to moderate upper limb severity subgroup (SAFE 5-8, n=101) that was assessed as impaired on each of the 10 possible impairments. | Percentage of the little to no upper limb severity subgroup (SAFE 9-10, n=302) that was assessed as impaired on each of the 10 possible impairments. |
| --- | --- | --- | --- | --- | --- |
| **0 impairments** | 30.5% (n=141) | 21.8% (n=35) | 18.3% (n=11) | 23.8% (n=24) | 35.1% (n=106) |
| **1 impairment** | 33.7% (n=156) | 30.4% (n=49) | 28.3% (n=17) | 31.7% (n=32) | 35.4% (n=107) |
| **2 impairments** | 16.8% (n=78) | 17.4% (n=28) | 15.0% (n=9) | 18.8% (n=19) | 16.6% (n=50) |
| **3 impairments** | 9.7% (n=45) | 11.8% (n=19) | 16.7% (n=10) | 8.9% (n=9) | 8.6% (n=26) |
| **4 impairments** | 6.3% (n=29) | 10.6% (n=17) | 13.3% (n=8) | 8.9% (n=9) | 4.0% (n=12) |
| **5 impairments** | 2.4% (n=11) | 6.2% (n=10) | 6.7% (n=4) | 5.9% (n=6) | 0.3% (n=1) |
| **6 impairments** | 0.4% (n=2) | 1.2% (n=2) | 1.7% (n=1) | 1.0% (n=1) | 0% (n=0) |
| **7 impairments** | 0.2% (n=1) | 0.6% (n=1) | 0% (n=0) | 1.0% (n=1) | 0% (n=0) |
| **8 impairments** | 0% (n=0) | 0% (n=0) | 0% (n=0) | 0% (n=0) | 0% (n=0) |
| **9 impairments** | 0% (n=0) | 0% (n=0) | 0% (n=0) | 0% (n=0) | 0% (n=0) |
| **10 impairments** | 0% (n=0) | 0% (n=0) | 0% (n=0) | 0% (n=0) | 0% (n=0) |
| **11 impairments** | 0% (n=0) | N/A | N/A | N/A | N/A |
